# Surgical complications and periprosthetic fractures following femoral impaction grafting in revision hip arthroplasty: a systematic review and meta-analysis of 4686 hips

**DOI:** 10.1007/s00264-026-06787-2

**Published:** 2026-04-01

**Authors:** Octavian Andronic, Rutenis Badikonis, Artsiom Klimko, Victor Yan Zhe Lu, Armando Hoch, Patrick O. Zingg

**Affiliations:** 1 Balgrist University Hospital, University of Zurich, Zurich, Switzerland; 2 University of Zurich, Zurich, Switzerland; 3 Kantonsspital Graubünden, Chur, Switzerland; 4 Chinese University of Hong Kong, Hong Kong, China

**Keywords:** Revision hip arthroplasty, Hip stem revision, Femoral reconstruction, Femoral bone loss, Graft incorporation, Impaction grafting

## Abstract

**Purpose:**

To evaluate the incidence of surgical complications, particularly periprosthetic fractures, following femoral impaction bone grafting (fIBG) in revision total hip arthroplasty (rTHA).

**Methods:**

This systematic review followed PRISMA guidelines. Studies reporting on fIBG in rTHA with a minimum follow-up of 12 months were included. Risk of bias was assessed using the Methodological Index for Non-Randomised Studies (MINORS). A random-effects meta-analysis with inverse-variance weighting was performed. Heterogeneity was assessed using Higgins and Thompson’s I^2^ statistic and Cochran’s Q test. Subgroup analyses were conducted based on patient characteristics, revision indication, follow-up period, bone loss severity, stem length, and cementation. Complications requiring intervention, as well as nerve lesions with partial or no recovery, were classified as major.

**Results:**

Forty-five studies including 4,686 hips (4,409 patients) were analyzed. The pooled incidence of major complications was 21% (*n* = 730; 95% CI 16–26%; I^2^ = 89%, *p* < 0.01). Of these, 78% required surgical intervention. The most common major complications were intraoperative fractures (22.7%), postoperative fractures (19.3%), and instability (19.2%). Higher complication rates were reported with long stems (44.1%) compared with short stems (18.8%, *p* = 0.016) and in cases of severe bone loss (Endo-Klinik grades III/IV, 25.3%) compared with I/II (14.2%, *p* = 0.028).

**Conclusion:**

Major complications following fIBG in rTHA are relatively frequent, although interpretation is limited by substantial study heterogeneity. Higher complication rates were observed in cases with severe bone loss and the use of long stems, likely reflecting greater surgical complexity.

## Introduction

**Fig. 1:**
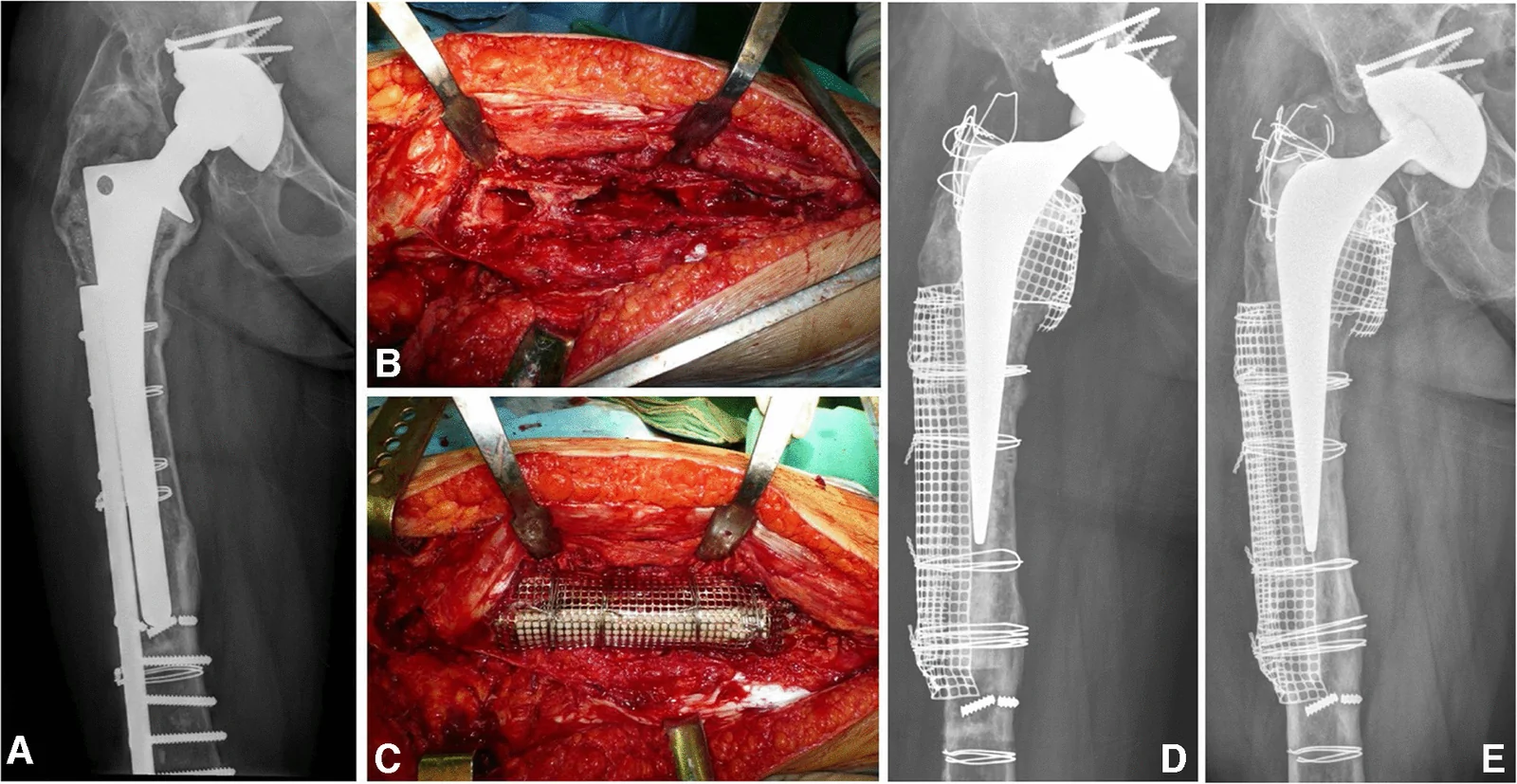
Case example of fIBG in rTHA. (**A**) 38-years-old female 19 years after THA with Anatomic Medullary Locking stem (DePuy, USA) by traffic accident. Severe stem loosening, subsidence, osteolysis, and Paprosky IV defect occurred. (**B**) The intraoperative photograph shows extensive cortical defects. (**C**, **D**) The hip was revised using Exeter V40 stem (Stryker, USA) with metal mesh and strut allograft and retaining original cup. (**E**) At last follow-up, successful consolidation of the allograft was achieved. *Used with permission of Elsevier, from *[5]

**Fig. 2:**
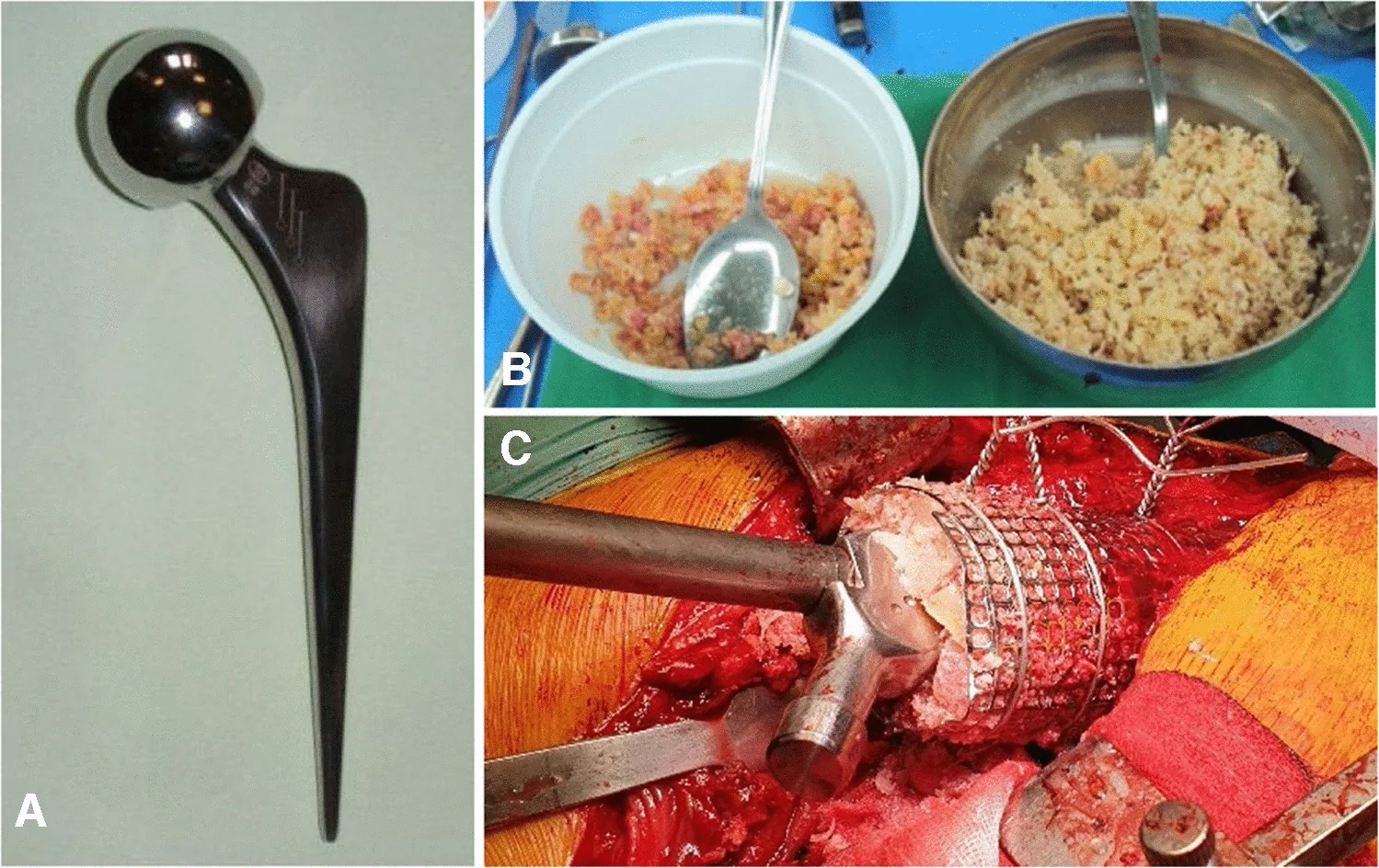
Operative technique of rTHA using fIBG with fresh frozen allo-femoral heads. (**A**) Exeter V40 stem. (**B**) Fresh frozen allo-femoral heads. (**C**) In case of a cortical defect, vigorous impaction bone grafting is performed by completely filling the femoral canal after changing the uncontained lesion with a contained lesion using a metal mesh. *Used with permission of Elsevier, from *[5]

Femoral impaction bone grafting (fIBG) is used in revision total hip arthroplasty (rTHA) to restore bone stock and provide stability when revising failed implants (Figs. 1 and 2) [1]. When performed correctly, it supports the femoral stem both biologically and mechanically [2]. Since its introduction in the early 1990s, fIBG has demonstrated promising clinical outcomes [3, 4]. However, concerns remain regarding complications such as stem subsidence, intraoperative fractures, and periprosthetic fractures, especially with longer operating times [5]. Outcomes vary across centres, reflecting differences in technique and patient selection [2, 6–9]. Although several studies have reported complication[6] and periprosthetic fracture rates with fIBG[10, 11], no comprehensive synthesis exists. This systematic review summarizes the available evidence on surgery-related complications and fracture incidence associated with fIBG in rTHA.

## Materials and methods

### Search Strategy

This systematic review followed PRISMA guidelines[12] and was registered in PROSPERO (CRD42024557047). A comprehensive search was conducted in CENTRAL, MEDLINE (PubMed), EMBASE, Scopus, AMED, and Web of Science from inception to June 30, 2024. Search terms included combinations of "impaction bone grafting", "revision hip arthroplasty", "femoral reconstruction", and "graft incorporation". Eligible studies involved adults undergoing femoral revision with fIBG and ≥ 12 months of follow-up.

### Selection and Data Extraction

Two reviewers (RB and AK) independently screened titles and abstracts, followed by full-text assessment. Disagreements were resolved by a third author (OA).

Inclusion criteria were original peer-reviewed human studies in English, level of evidence ≥ IV according to the Oxford Centre for Evidence-Based Medicine 2011 Levels of Evidence[13], reporting outcomes of fIBG in rTHA. Exclusion criteria included non-English studies, reviews, technical notes, animal or cadaveric studies, overlapping populations, < 10 patients, follow-up < 1-year, primary THA with fIBG, concomitant distal femoral arthroplasty, cancer patients, rheumatoid or genetic conditions, cost-effectiveness or technical-only studies, custom compaction devices, and studies without DOIs.

### Risk of Bias

Bias was assessed using the MINORS tool, evaluating 8 items for noncomparative and 12 for comparative studies [14]. Scores ranged from 0 to 16 (noncomparative) or 24 (comparative) [15]. Two authors independently assessed risk of bias, resolving differences by consensus with a senior author.

### Meta-Analysis

Quantitative data (e.g., rates of major and minor complications) were pooled where comparable data were available using the “metafor” package in R (R Core Team, R Foundation for Statistical Computing, Vienna, Austria) [16]. Binary outcomes were analyzed using random-effects models with inverse-variance weighting. Heterogeneity was assessed via Higgins and Thompson's I^2^ and Cochran’s Q test[17, 18], with prediction intervals reported. Standard deviations were estimated using the Wan et al. estimator[19] or derived from *p*-values according to the Cochrane Handbook [20]. Heterogeneity was defined as moderate (I^2^ > 40%) or high (I^2^ > 75%) [21].

Complications were grouped into major (e.g., operatively managed fractures, instability, nerve damage without recovery) and minor (e.g., conservatively managed fractures, reversible nerve damage) (Table 1). Component-related complications were divided into subsidence and other complications; subsidence requiring revision was classified as major.

**Table 1:** Classification of minor and major complications

Minor Complications (*n* = 488)	Major Complications (*n* = 730)
Intraoperative trochanteric fractures/perforations/cracks without intervention (44%)	Intraoperative fractures requiring additional intervention (inclusive all intraoperative shaft fractures) (23%)
Heterotopic ossifications without intervention (36%)	Postoperative fractures requiring intervention at any stage (19%)
Trochanteric complications (nonunion, bursitis etc.) without intervention (7%)	Instability (19%)
Superficial infections/hematomas without intervention (5%)	Deep infections (15%)
Postoperative fractures with conservative treatment (4%)	Component-related complications requiring revision at any stage (stem fractures, aseptic loosening, acetabular loosening etc.) (15%)
Component-related complications without revision (incl. acetabular loosening) (2%)	Stem subsidence requiring intervention at any stage (5%)
Nerve damage with full recovery (1%)	Nerve damage with partial or no recovery (2%)
	Superficial infections/hematomas requiring intervention at any stage (1%)
	Trochanteric complications (nonunion, bursitis etc.) requiring intervention at any stage (1%)
	Heterotopic ossifications requiring intervention at any stage (< 1%)

The Endo-Klinik[58] and Paprosky[59] bone loss classifications were merged into mild/moderate (grades I–II) and severe (grades III–IV).

Subgroup analyses were performed in RStudio 4.2.1 for complication rates and fractures. Continuous data were pooled using the *metacont* function and proportions using *metaprop*. The Wan et al. estimator[19] was used to determine the standard deviation if it was not provided. Random-effects models were used for all analyses due to anticipated heterogeneity. Statistical significance was set at *p* < 0.05. The I^2^ statistic and Cochran’s Q test[17, 18] were used to determine heterogeneity. Subgroup analyses were conducted based on the predominant study characteristics: gender, age (< 65/≥ 65 years), revision indication (infection/aseptic loosening), follow-up (< 5/≥ 5 years), bone loss severity (grades I–II/III–IV), stem length (short/long), and cementation status.

## Results

**Fig. 3:**
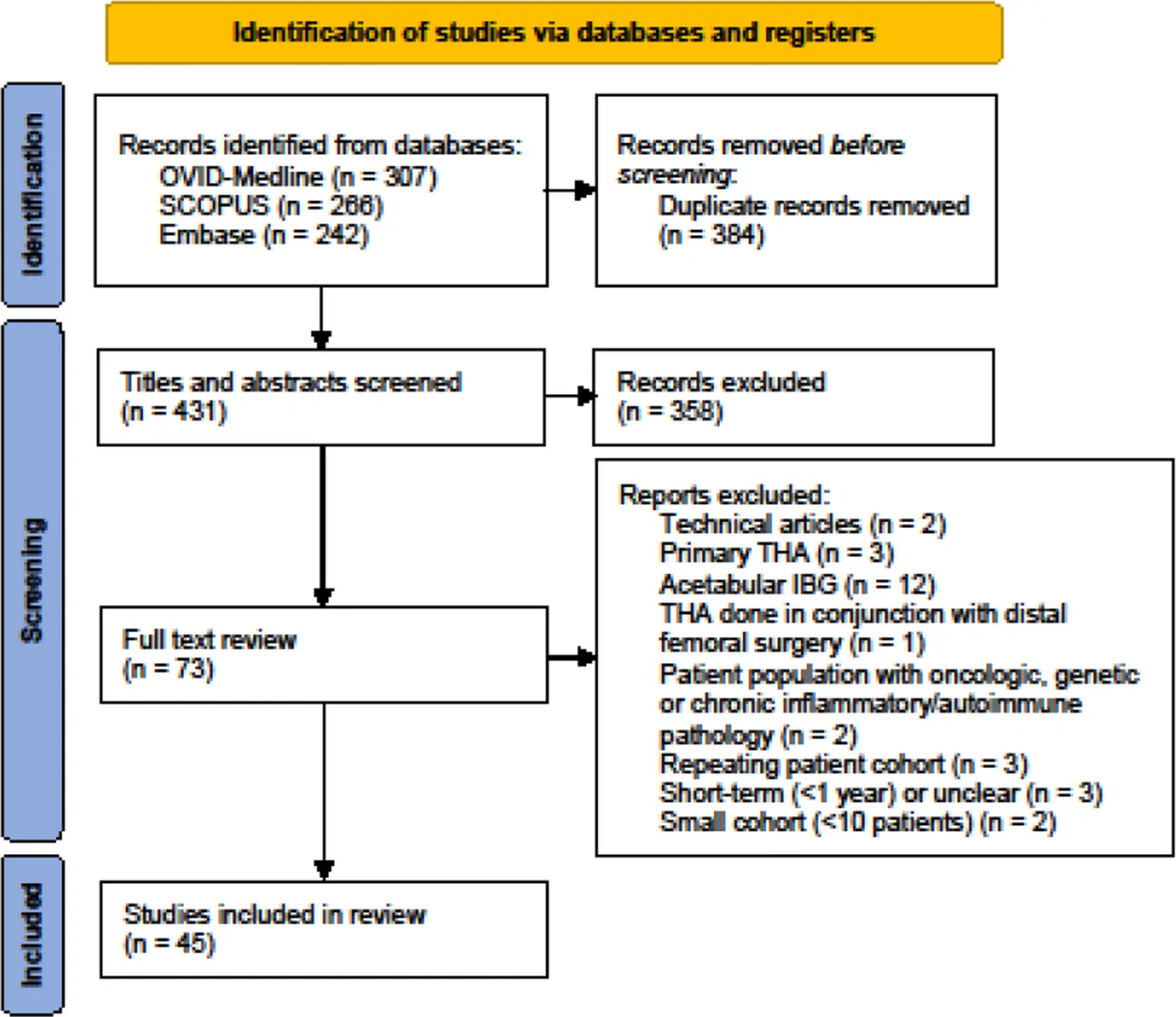
PRISMA systematic search flowchart

The initial database search yielded 1,215 records. After removal of 384 duplicates, 831 records remained for screening (Fig. 3). Title and abstract review excluded 358 articles, leaving 73 full texts for assessment. Of these, 45 studies met the inclusion criteria and were included in the final review.

### Database Search

Included studies were retrospective with MINORS scores ranging from 10 to 18. Most were classified as evidence level III or IV. According to the MINORS protocol, common methodological limitations included a lack of comparator groups (all except Wimmer et al.[26]), no collection of prospective data or sample size calculation (all studies), and inconsistent reporting of follow-up or loss to follow-up [7, 11, 25, 26, 35, 36, 40–42, 44, 46, 48, 60].

### Population characteristics

A total of 4,686 hips in 4,409 patients undergoing fIBG were included (Table 2). In one study[45], rTHA was performed concomitantly with femoral and acetabular IBG, with some patients receiving acetabular IBG only. As complications specific to the fIBG group could be extracted, the study was retained. Demographic analyses were based on the total study population. In seven studies[7, 11, 34, 36, 38, 47, 60] patient numbers reflected initial enrolment rather than final follow-up; therefore, epidemiological data in Table 2 refer to total enrolled patients rather than only those undergoing fIBG with complete follow-up.

**Table 2:** Summary of study demographics (number of patients, gender distribution, mean age and length of follow-up), as well as revision indications, stem length, cementation and bone loss grade for each study

Author and Year	Number of Patients in Study	Number of Hips in Study	Mean Age (Range)	Gender	Mean Follow-up in Years (Range)	Indications for Revision	Stem Length	Cementation	Bone Loss Grade
Bunting et al., 2023[22]	29	29	61 (43–84)	21 M, 8 F	7.42 (0.67–13.92)	Infection	Standard: 130 mm (62%), 180 mm (27.5%); Long: 200, 260 mm	Cemented	Paprosky—Grade I: 4, Grade II: 14, Grade IIIA: 8, Grade IIIB: 1, Grade IV: 2
Iwase et al., 2023[23]	93	99	66.3 (36–84)	21 M, 72 F	11 (2–23.8)	Aseptic loosening, reconstruction after infection, femoral osteolysis, periprosthetic fracture	Not specified	Cemented	Endo-Klinik—Grades I-IV
Park et al., 2022[5]	13	13	50.2 (22–77)	7 M, 6 F	11.1 (5.3–15.1)	Paprosky IV femoral bone defects	Not specified	Cemented	Paprosky—Grade IV: 4
Stigbrand et al., 2017[11]	67	69	69 (49.6–88.4)	40 M, 29 F	7 (0.73–13.27)	Aseptic loosening, septic loosening	Standard: 170 mm (64 cases); Long: 150 mm (5 cases)	Cemented	Endo-Klinik—Grade II: 7, Grade III: 46, Grade IV: 16
Wilson et al., 2016[24]	631	705	69.7 (22–95)	296 M, 335 F	14.7 (9.8–28.3)	Aseptic loosening, periprosthetic fracture, infection, malposition, stem fracture, cement-in-cement exchange	Standard: 150 mm; Long: 200–260 mm; Short: 125 mm	Cemented	Not specified
Garvin et al., 2013[25]	71	78	67 (33–84)	39 M, 32 F	10.6 (2–19)	Mechanical loosening, lack of femoral cancellous bone	Standard: 120–140 mm; Long: 175–225 mm	Cemented	Endo-Klinik—Grade I: 0, Grade II: 22, Grade III: 42, Grade IV: 14
Wimmer et al., 2013[26]	68	70	69.2 (49.8–88.6)	23 M, 47 F	4.4 (NA)	Aseptic loosening, periprosthetic fracture, infection, shaft fissure, avulsion of greater trochanter	Not specified	Uncemented	Paprosky—Grade I: 4, Grade IIA: 20, Grade IIB: 15, Grade IIC: 12, Grade III: 19
Ten Have et al., 2012[27]	29	31	65 (35–82)	6 M, 23 F	12.6 (10–14.7)	Aseptic loosening, periprosthetic fracture, Girdlestone excision arthroplasty, mid-thigh pain	Standard: 150 mm	Cemented	Endo-Klinik—Grade I: 1, Grade II: 10, Grade III: 9, Grade IV: 11
Howie et al., 2010[28]	53	56	63 (27–88)	42 M, 14 F	NA (2–15)	Aseptic loosening, infection, periprosthetic fracture, femoral stem fracture	Standard and Long-length stems	Cemented	Endo-Klinik—Grades I-IV
Buttaro et al., 2009[29]	14	15	67 (43–82)	1 M, 14 F	3.6 (1.67–6)	Septic failure, aseptic loosening, periprosthetic fracture	Long: 222 mm (130–260 mm)	Cemented	Paprosky—Grade IV: 4, Grade IIIB: 1
Hassaballa et al., 2009[30]	68	69	64.2 (34–88)	37 M, 32 F	2.37 (1–7.42)	Bone stock deficiency	Not specified	Cemented	Endo-Klinik—Grade I: 11, Grade II: 28, Grade III: 30
Ornstein et al., 2009[4]	1188	1305	71 (29–94)	538 M, 650 F	NA (5–18)	Aseptic loosening, infection, subsidence, fracture, pseudarthrosis, dislocation, stem fracture	Standard: 1081 hips; Long: 205 mm or longer in 145 hips	Cemented	Not specified
Sierra et al., 2008[31]	40	42	73.8 (49–89)	14 M, 26 F	7.5 (NA)	Aseptic loosening, periprosthetic fracture, nonunion, infection	Long: 220–260 mm	Cemented	Endo-Klinik—Grade I: 2, Grade II: 4, Grade III: 25, Grade IV: 9
Wraighte and Howard, 2008[3]	75	75	68 (35–87)	40 M, 35 F	10.5 (6.3–14.1)	Aseptic loosening, periprosthetic fracture, infection	Not specified	Cemented	Endo-Klinik—Grade I: 2, Grade II: 19, Grade III: 50, Grade IV: 4
Mahoney et al., 2005[32]	41	43	67.2 (34–84)	22 M, 18 F	4.7 (2.0–7.7)	Aseptic loosening	Standard: 39; Long: 4	Cemented	Endo-Klinik—Grade II: 16, Grade III: 24, Grade IV: 4
Ornstein et al., 2004[33]	14	15	74 (60–82)	7 M, 7 F	5 (NA)	Aseptic loosening	Standard	Cemented	Gustilo and Pasternak—Type I: 7, Type II: 6, Type III: 2
Halliday et al., 2003[34]	202	221	68 (35–89)	91 M, 116 F	8.1 (5–16)	Aseptic loosening, femoral fracture	Long: 150–260 mm	Cemented	Endo-Klinik—Grade I: 12, Grade II: 106, Grade III: 62, Grade IV: 6
Gore et al., 2002[35]	25	26	68 (34–89)	9 M, 17 F	NA (0–6)	Degenerative joint disease, avascular necrosis, rheumatoid arthritis, femoral neck fracture	Collarless, polished, tapered stem from cobalt chrome alloy	Cemented	Not specified
Lind et al., 2002[36]	74	74	NA	NA	3.6 (1–7)	Aseptic loosening, septic loosening	Not specified	Cemented	Endo-Klinik—Grade I: 7, Grade II: 34, Grade III: 39, Grade IV: 7; Mallory classification—Grades I-IIIc
Robinson et al., 2002[37]	53	57	63.7 (31.7–87.6)	29 M, 24 F	2.25 (0.5–5.67)	Aseptic loosening, septic loosening, periprosthetic fracture	Standard: 150 mm	Cemented	Endo-Klinik—Grade I: 8, Grade II: 22, Grade III: 27, Grade IV: 0
Piccaluga et al., 2002[38]	57	58	65.2 (32–88)	22 M, 35 F	4.7 (2.0–7.7)	Aseptic loosening	Not specified	Cemented	Endo-Klinik—Grade II: 9, Grade III: 41, Grade IV: 9
van Biezen et al., 2000[39]	21	21	65 (33–82)	3 M, 18 F	5 (3.42–7.08)	Aseptic loosening, femoral fracture, painful prosthesis, Girdlestone situation	Standard: 150 mm	Cemented	Endo-Klinik—Grade III or IV
Pekkarinen et al., 2000[6]	65	68	71 (40–88)	28 M, 37 F	3 (0.08–6)	Aseptic loosening, infected THR, dislocation	Standard	Cemented: 36, Uncemented: 32	Endo-Klinik—Grade I: 8, Grade II: 22, Grade III: 25, Grade IV: 13
Flugsrud et al., 2000[9]	10	10	74 (65–82)	5 M, 5 F	4 (3.0–4.6)	Not specified	Not specified	Cemented	Paprosky—Grade II: 1, Grade III: 9
Kärrholm et al., 1999[40]	24	24	65 (38–84)	10 M, 14 F	3 (up to 3)	Femoral stem loosening	Standard: 135 mm	Cemented	Gustilo and Pasternak classification—Type I: 3, Type II: 17, Type III: 4; Endo-Klinik—Grade I: 1, Grade II: 16, Grade III: 7
Eldridge et al., 1997[7]	78	79	68 (27–88)	41 M, 38 F	1.05 (0.5–2.75)	Aseptic loosening	Not specified	Cemented	Endo-Klinik—Grade I: 1, Grade II: 16, Grade III: 7
Masterson et al., 1997[41]	35	35	62 (27–80)	16 M, 19 F	0.5 (0.5)	Aseptic loosening, two-stage revision for infection, thigh pain	Not specified	Cemented	Endo-Klinik—Grade II: 6, Grade III: 22, Grade IV: 7
Gie et al., 1993[42]	68	56	68.9 (46–87)	32 M, 24 F	2.5 (1.5–4.08)	Femoral component loosening, poor bone stock	Standard	Cemented	Endo-Klinik—Grade I: 3, Grade II: 40, Grade III: 13
Deakin and Bannister, 2007[43]	85	59	69 (NA)	42 M, 43 F	3.7 (0.5–11)	Aseptic loosening, infection, periprosthetic fracture, femoral stem fracture	Standard and long	Cemented	Endo-Klinik—Grade I: 9, Grade II: 24, Grade III: 26
de Roeck and Drabu, 2001[44]	32	30	70 (47–85)	8 M, 24 F	4 (NA)	Aseptic loosening, septic loosening, fractured acetabular cup	Not specified	Cemented	Not specified
Edwards et al., 2003[45]	53	53	65.7 (26–83)	32% M, 68% F	3.1 (1.5–5.2)	Aseptic loosening	Not specified	Cemented	Endo-Klinik—Grade I: 3, Grade II: 9, Grade III: 19, Grade IV: 7
Fetzer et al., 2001[8]	26	26	69.3 (48.1–85.4)	13 M, 13 F	6 (4.0–8.1)	Aseptic loosening	Not specified	Cemented	Paprosky—IIIA: 21, IIIB: 5
Kerboull et al., 2008[46]	129	129	64 (43–79)	59 M, 70 F	8.4 (2–16)	Aseptic loosening, septic loosening	Not specified	Cemented	Endo-Klinik—Grade II: 75, Grade III: 43, Grade IV: 11
Knight and Helming, 2000[47]	30	30	70 (42–97)	17 M, 14 F	2.6 (0.6–3.8)	Aseptic loosening, osteolysis, thigh pain, periprosthetic fracture, acetabular component loosening	Not specified	Cemented	Not specified
Lamberton et al., 2011[2]	487	540	69.7 (31–95)	243 M, 297 F	6.7 (2–15)	Aseptic loosening, infection, periprosthetic fracture	Standard: 473; Long: 67 (200–260 mm)	Cemented	Not specified
Mikhail et al., 1999[48]	40	43	59 (47–84)	22 M, 18 F	6 (5–7)	Aseptic failure (mechanical)	Not specified	Cemented	Endo-Klinik—Grade II: 13, Grade III: 23, Grade IV: 7
Oshima et al., 2012[49]	54	55	70 (49–88)	12 M, 42 F	7.7 (3–12.5)	Osteoarthritis, osteonecrosis, femoral neck fracture, rheumatoid arthritis	Not specified	Cemented	Endo-Klinik—Grade I: 3, Grade II: 24, Grade III: 28
Robinson et al., 2002[50]	19	19	59 (30–85)	12 M, 7 F	2.75 (1–5.67)	Aseptic loosening, septic loosening	Not specified	Cemented	Endo-Klinik—Grade I: 5, Grade II: 6, Grade III: 8
Schreurs et al., 2005[51]	33	33	63 (33–82)	9 M, 24 F	10.4 (8–13)	Aseptic loosening, septic loosening	Not specified	Cemented	Endo-Klinik—Grade I: 3, Grade II: 14, Grade III: 12, Grade IV: 4
Singh and Bhalodiya, 2013[52]	48	53	59 (44–68)	42 M, 6 F	5.5 (NA)	Aseptic osteolysis, periprosthetic fracture, broken femoral stem, septic loosening, comminuted fracture	Long (190–385 mm)	Uncemented	Paprosky—Grade IIA: 1, Grade IIIA: 32, Grade IIIB: 5
Sotiriou et al., 2020[53]	54	55	68 (46.66–89.34)	24 M, 20 F	NA (2–16)	Periprosthetic joint infection	Standard (Lubinus SP II stem)	Cemented	Endo-Klinik—Grade 0: 41, Grade II: 0, Grade III: 5, Grade IV: 1
Park et al., 2023[54]	43	47	55 (39–75)	37 M, 10 F	13.5 (10.9–17.8)	Aseptic loosening, septic loosening, periprosthetic fracture	Standard	Cemented	Endo-Klinik—Grade II: 12, Grade III: 16, Grade IV: 11
te Stroet et al., 2014[55]	24	24	71 (39–86)	10 M, 14 F	8.28 (0.04–15.7)	Aseptic loosening, septic loosening, dislocation, chronic pain, periprosthetic fracture	Long: 205–220 mm	Cemented	Endo-Klinik—Grade II: 7, Grade III: 13, Grade IV: 4
Verspeek et al., 2021[56]	33	33	46 (30.32–61.68)	14 M, 19 F	17 (12–22)	Aseptic loosening, septic loosening	Not specified	Cemented	Della Valle—Type I: 7, Type II: 17, Type IIIA: 6, Type IIIB: 1, Type IV: 2
Yan et al., 2010[57]	13	15	61 (38–84)	4 M, 9 F	7.7 (4–13)	Aseptic loosening, infection	Standard	Cemented	Endo-Klinik—Grade II: 4, Grade III: 7, Grade IV: 4

Female patients comprised 54%, and the weighted mean age across studies was 66 years (range 22–95). Paprosky classification was used in 16% (7/45) and Endo-Klinik classification in 67% (30/45) of studies, while 18% (8/45) did not use any defect classification. Among studies that did, grade III–IV defects predominated (60% with Paprosky and 55% with Endo-Klinik).

Standard-length stems (~ 150 mm) were used in 64% (29/45) of studies. Long stems (200–260 mm) were used in 29% (13/45), particularly in higher-grade defects. Cemented femoral fixation was reported in 93% (42/45) of studies, while one study[6] used both cemented and cementless stems.

Supplementary fixation devices were reported in 71% (32/45) of studies, most commonly cerclage wires (71%), followed by metal meshes (40%), cortical struts (36%), and plates or gauze meshes (20%), typically in cases with severe femoral bone loss (Endo-Klinik grades III/IV).

### Complications

All 45 studies (100%, 4,409 patients and 4,686 hips) reported complications (Table 3). Two studies (4%, 2/45)[7, 30] focused exclusively on subsidence and included no other complication data. In total, 1,218 complications were reported, of which 60% (730) were major and 40% (488) were minor. The most frequent major complications were intraoperative fractures requiring intervention (23%, 166/730), postoperative fractures requiring intervention (19%, 141/730), and instability (19%, 140/730). The most common minor complications were intraoperative trochanteric fractures, perforations, or cracks (44%, 214/488), heterotopic ossifications (36%, 177/488), and trochanteric non-unions or bursitis (7%, 36/488). Instability occurred in 140 patients; 36% (50/140) underwent or were scheduled for surgery, while 41% (58/140) were treated with closed reduction. Surgical treatment most commonly involved acetabular revision (30%, 15/50), followed by combined cup and stem revision (10%, 5/50), and isolated component revisions. Remaining cases of instability were not further specified.

**Table 3:** Data of complications (minor/major) and periprosthetic fractures (intraoperative/postoperative) for each study

Study	Number of Patients in Study	Total Number of Complications	Minor Complications	Major Complications	Intraoperative Fractures (Type/Treatment)	Postoperative Fractures (Onset Time Postoperatively in Months/Treatment)
Bunting et al., 2023	29	12	0	12	0	0
Iwase et al., 2023	93	15	0	15	0	8 (5 Fractures: < 26, 3 Fractures: 136—152/1 Revision, 7 ORIF)
Park et al., 2022	13	1	0	1	0	1 (132/1 ORIF)
Stigbrand et al., 2017	67	9	0	9	0	3 (Not specified/3 ORIF)
Wilson et al., 2016	631	240	120	120	116 (Shaft: 15, Trochanteric: 19, Perforation: 44, Calcar crack: 26, Other: 12/Not specified)	23 (Not specified/11 Revisions, 12 Revisions + ORIF)
Garvin et al., 2013	71	11	1	10	3 (Not specified/1 Revision with long stem, 2 Wires fixations)	3 (Not specified/1 Revision with long stem, 1 ORIF, 1 Conservative)
Wimmer et al., 2013	68	15	5	10	7 (Shaft: 1, Trochanteric: 1, Perforation: 2, Calcar crack: 3, Other: 0/1 ORIF, 1 Wires fixation, Others not specified)	1 (62/1 Revision)
Ten Have et al., 2012	29	10	2	8	0	4 (3 Fractures: < 6, 1 Fracture: 72/3 Reoperations, not specified, 1 Conservative)
Howie et al., 2010	53	25	1	24	9 (Shaft: 6, Trochanteric: 0, Perforation: 3, Calcar crack: 0, Other: 0/9 Wires fixations)	3 (1 Fracture: 11, 2 fractures: 3, 1 fracture: 96/2 ORIF, 1 Conservative)
Buttaro et al., 2009	14	21	10	11	0	2 (31, 48/1 Revision with alloprosthesis (+ Rerevision with alloprosthesis), 1 Revision with long stem)
Hassaballa et al., 2009	68	6	0	6	0	0
Ornstein et al., 2009	1188	70	0	70	0	17 (< 48/17 Revisions)
Sierra et al., 2008	40	22	2	20	6 (Shaft: 0, Trochanteric: 0, Perforation: 6, Calcar crack: 0, Other: 0/Wires and mesh-/strut-grafts fixations, no numerical data)	2 (3, 12/2 ORIF)
Wraighte and Howard, 2008	75	15	2	13	5 (Shaft: 3, Trochanteric: 2, Perforation: 0, Calcar crack: 0, Other: 0/5 Wires fixations)	2 (1, 27/1 Revision, 1 ORIF)
Mahoney et al., 2005	41	7	3	4	3 (Not specified/2 Revisions with long stems, Other not specified)	1 (2/1 Conservative)
Ornstein et al., 2004	14	4	0	4	0	2 (1, 3/2 ORIF)
Halliday et al., 2003	202	133	107	26	17 (Not specified/1 Wires fixation, 4 ORIF, 1 Wires fixation + ORIF, Others not specified)	9 (4, 18, 27, 74, 103, 4 Fractures: Not specified/5 ORIF, 4 Conservative)
Gore et al., 2002	25	8	2	6	1 (Not specified; Treatment: 1 Wires fixation)	3 (2 Fractures: 1, 1 Fracture: 2/3 Reoperations, Not specified)
Lind et al., 2002	74	22	11	28	17 (Not specified/17 Wires fixations/Metal meshes)	2 (< 30/2 ORIF)
Robinson et al., 2002	53	18	2	16	5 (Shaft: 1, Trochanteric: 3, Perforation: 0, Calcar crack: 0, Other: 1/3 Wires fixations, Others not specified)	3 (0, 7, 64/3 ORIF)
Piccaluga et al., 2002	57	41	34	7	1 (Not specified/1 Wires fixation + Strut-graft)	0
van Biezen et al., 2000	21	5	2	3	0	2 (3, 5/1 Reoperation, Not specified, 1 Conservative)
Pekkarinen et al., 2000	65	33	2	31	17 (Shaft: 9, Trochanteric: 8, Perforation: 0, Calcar crack: 0, Other: 0/15 Additional fixations, Not specified)	6 (1 Fracture: 4, 5 Fractures: Not specified/6 Reoperations, Not specified)
Flugsrud et al., 2000	10	10	9	1	0	0
Kärrholm et al., 1999	24	4	2	2	0	1 (36/1 ORIF)
Eldridge et al., 1997	78	0	0	8	0	0
Masterson et al., 1997	35	9	1	8	4 (Not specified/4 Wires fixations)	2 (2 Fractures: 2/2 Cortical onlay allografts)
Gie et al., 1993	68	7	1	6	3 (Perforation: 1, Others not specified/2 ORIF with plates, Others not specified)	1 (9/1 ORIF with two-time plating with allograft)
Deakin and Bannister, 2007	85	9	1	8	3 (Not specified/3 Revisions)	1 (60/1 Revision)
de Roeck and Drabu, 2001	32	15	9	6	0	1 (Not specified/1 Reoperation, Not specified)
Edwards et al., 2003	53	6	1	5	3 (Perforation: 1, Others not specified/2 ORIF with plates, 1 Strut-graft)	1 (1/1 Conservative)
Fetzer et al., 2001	26	23	19	4	0	3 (2, 9, 21/1 Reoperation, Not specified, 2 Conservative)
Kerboull et al., 2008	129	7	3	4	1 (Not specified/1 Wires fixation)	0
Knight and Helming, 2000	30	20	4	16	10 (Shaft: 6, Trochanteric: 0, Perforation: 0, Calcar crack: 4, Other: 0/5 Wires fixations, 1 Wires fixation + ORIF with plate, 1 Revision, 2 Revisions + Wires fixations + ½ Strut-graft)	2 (Not specified/2 Wires fixations + Strut-grafts)
Lamberton et al., 2011	487	199	111	88	107 (Shaft: 10, Trochanteric: 19, Perforation: 46, Calcar crack: 32, Other: 0/Not specified)	29 (11 Fractures: < 12, 7 Fractures: > 4, 10 Fractures: > 24/4 Revisions, 18 ORIF, 7 Conservative)
Mikhail et al., 1999	40	13	0	13	0	2 (Not specified/2 ORIF)
Oshima et al., 2012	54	22	0	22	8 (Shaft: 0, Trochanteric: 0, Perforation: 0, Calcar crack: 8, Other: 0/7 Wires fixations, 1 ORIF with plate)	5 (Not specified/5 ORIF (+ 1 Rerevision with ORIF, 1 Rerevision with long stem)
Robinson et al., 2002	19	7	0	7	1 (Not specified/1 Wires fixation)	2 (6, 9/1 Revision, 1 ORIF)
Schreurs et al., 2005	33	22	18	4	3 (Perforation: 2, Others not specified/3 Conservative)	3 (3, 6, 22/3 ORIF)
Singh and Bhalodiya, 2013	48	23	0	23	12 (Shaft: 0, Trochanteric: 12, Perforation: 0, Calcar crack: 0, Other: 0/12 Wires fixations)	0
Sotiriou et al., 2020	54	11	1	10	0	5 (Not specified/5 Prosthesis-retaining revisions)
Park et al., 2023	43	8	0	8	8 (Shaft: 4, Trochanteric: 0, Perforation: 0, Calcar crack: 4, Other: 0/4 Wires fixations, 4 Wires fixations + Strut-grafts)	0
te Stroet et al., 2014	24	15	0	15	5 (Not specified/5 Wires fixations)	2 (Not specified/2 ORIF)
Verspeek et al., 2021	33	11	0	11	0	1 (Not specified/1 Cup revision (+ 1 Rerevision of both components))
Yan et al., 2010	13	9	2	7	5 (Shaft: 3, Trochanteric: 0, Perforation: 2, Calcar crack: 0, Other: 0/Not specified)	2 (5, 22/1 Revision, 1 ORIF)

Deep infection occurred in 109 patients, with 78% (85/109) undergoing surgical revision and 3% (3/109) treated with suppressive antibiotics. Superficial infections or haematomas were reported in 34 patients; 26% (9/34) required surgical treatment.

Thirty-seven revisions for symptomatic subsidence were reported. Overall, 117 component-related adverse events were identified, most commonly aseptic loosening (56%, 65/117), followed by cement-in-cement stem exchange (16%, 19/117), implant failure (10%, 12/117), non-union with pseudoarthrosis (8%, 9/117), and malalignment (5%, 6/117). Most (91%, 106/117) required surgical management.

Trochanteric complications occurred in 44 patients, including trochanteric bursitis (14%, 6/44), all requiring intervention, and trochanteric non-union (86%, 38/44), which was usually managed conservatively (95%, 36/38). Three non-unions reported by Piccaluga et al.[38] were secondary to dislocation and were therefore categorized under instability.

Heterotopic ossifications occurred in 178 patients, with only one case requiring surgical excision. Nerve injuries occurred in 19 patients; 32% (6/19) recovered fully, while 68% (13/19) reported persistent neurological symptoms at final follow-up.

All studies reported intraoperative and/or postoperative fractures when present (Table 3**)**. Four studies (9%, 4/45)[7, 9, 22, 30] reported no fractures. Intraoperative fractures occurred in 380 cases, most commonly cortical perforations (28%, 107/380), calcar fractures (20%, 77/380), trochanteric fractures (17%, 64/380), and shaft fractures (15%, 58/380). Additional intervention was required in 33% (126/380), most often cerclage fixation (75%, 95/126).

Postoperative periprosthetic fractures were reported in 160 patients. Timing was documented in 54% (86/160), most commonly occurring > 12 months postoperatively (43%, 37/86). Aetiology was reported in 39% (63/160), with traumatic events (54%), technical error (33%), and stress fractures (13%) being most common. Most postoperative fractures (88%, 141/160) required surgical treatment, including open reduction and internal fixation (44%, 62/141), prosthesis revision (30%, 42/141), or combined procedures (9%, 12/141). Conservative management was reported in 13% (19/160). Because the Vancouver classification was used in only two studies[23, 28], fracture morphology could not be analyzed in detail.

### Meta-Analyses

#### Complications

**Fig. 4:**
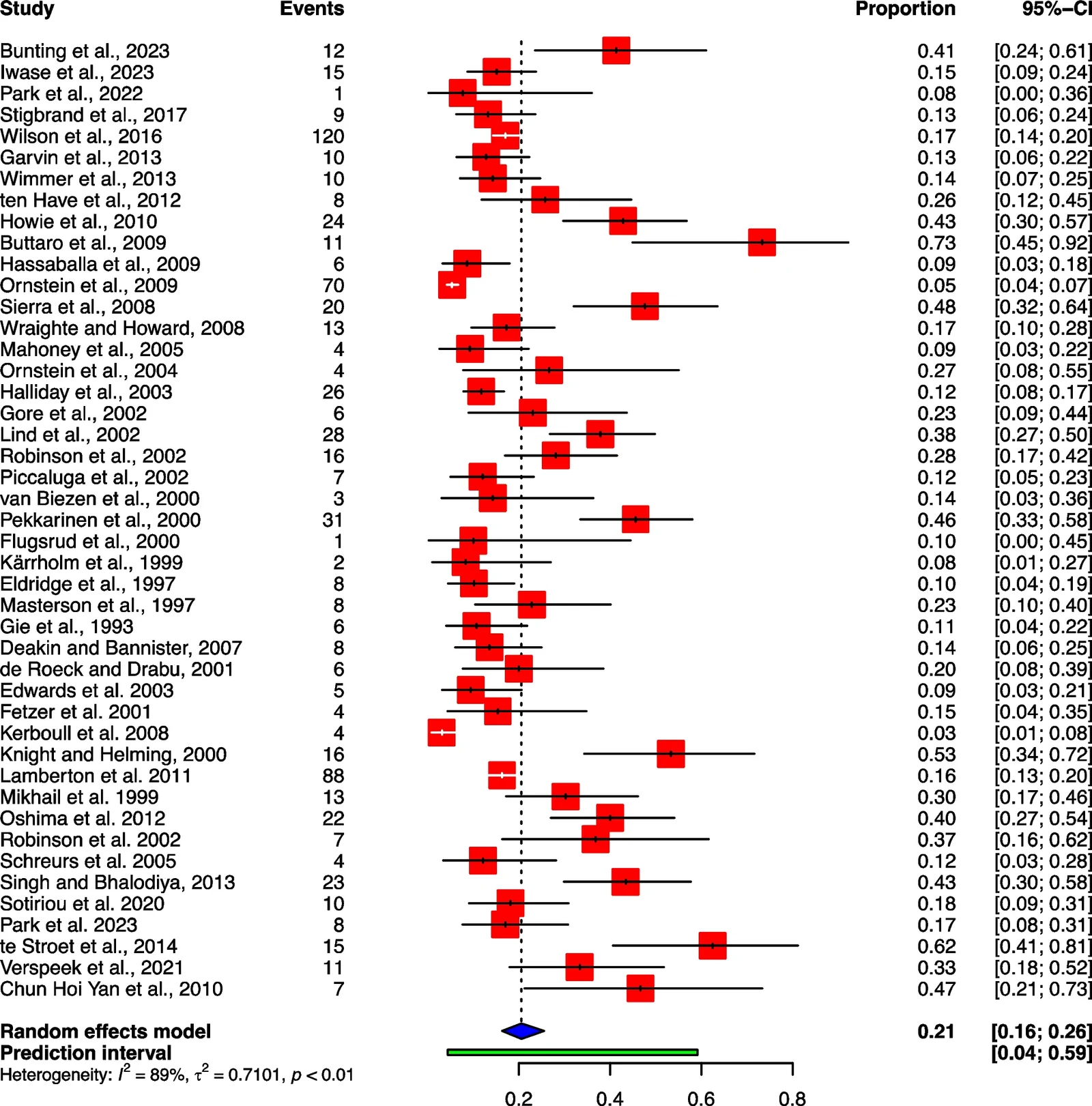
Pooled analysis of major complications. The forest plot displays the proportion of major complications in each study

The pooled incidence of major complications was 21% (95% CI: 16–26%) with high heterogeneity (I^2^ = 89%, *p* < 0.01, Fig. 4) and with 78% (573/730) of these requiring surgical intervention. Longer stems were usually used in cases involving more extensive femoral bone loss or structural defects, reflecting higher baseline surgical complexity. Major complications were significantly more frequent when using long-stem prostheses (44.1%, 95% CI: 17.4–74.7%) compared to standard stems (18.8%, 95% CI: 13.6–25.4%, *p* = 0.016) (Table 4), and with the Endo-Klinik classification grade III/IV defects (25.3%, 95% CI: 6.0–22.6%) compared to grade I/II defects (14.2%, 95% CI: 16.4–32.6%, *p* = 0.028). No significant difference was found based on the Paprosky classification (*p* = 0.892). There was a noticeable trend towards a higher complication incidence with infection as the indication for surgery (26.6%, 95% CI: 6.7–64.6%) compared to aseptic indications (19.9%, 95% CI: 14.5–23.8%), although this was not statistically significant (*p* = 0.533).

**Table 4:** Subgroup analysis of pooled major complications

Variable	Number of Studies	Proportion	95% Confidence Interval	*p*-value
**Gender**				0.805
Male	21	0.213	0.128–0.263	
Female	24	0.202	0.141–0.281	
**Age**				0.390
≥ 65 years	30	0.189	0.148–0.344	
< 65 years	14	0.232	0.131–0.240	
**Indication**				0.533
Infection	5	0.266	0.067–0.646	
Aseptic Loosening	40	0.199	0.145–0.238	
**Follow-up**				0.816
≥ 5 years	25	0.210	0.153–0.282	
< 5 years	20	0.200	0.111–0.259	
**Paprosky**				0.892
Grades I + II	2	0.240	0.000–0.997	
Grades III + IV	5	0.261	0.060–0.663	
**Endo-Klinik**				**0.028**
Grades I + II	11	0.142	0.164–0.326	
Grades III + IV	15	0.253	0.060–0.226	
**Stem**				**0.016**
Short	19	0.188	0.136–0.254	
Long	5	0.441	0.174–0.747	
**Cemented**				0.544
Yes	43	0.203	0.147–0.244	
No	2	0.264	0.003–0.997	

**Fig. 5:**
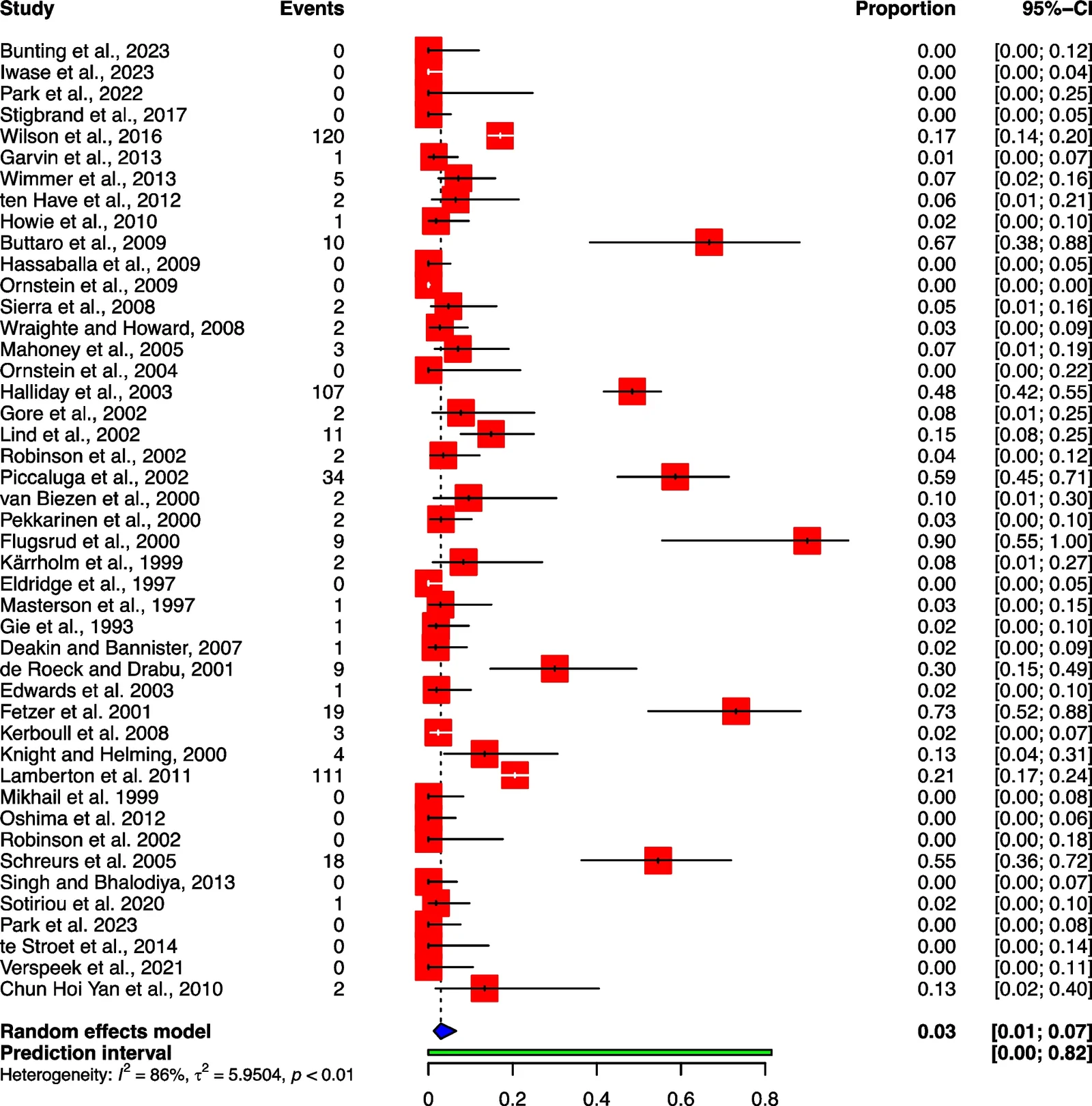
Pooled analysis of minor complications. The forest plot displays the proportion of minor complications in each study

The pooled incidence of minor complications was 3% (95% CI: 1–7%, I^2^ = 86%), with high heterogeneity between studies (I^2^ = 86%, *p* < 0.01, Fig. 5). A number of studies [4, 5, 7, 11, 22, 23, 33, 48–50, 52, 54–56, 61] reported perfect results with no minor complications, achieving a complication rate of 0%. Conversely, other studies demonstrated a higher incidence of complications, with the study by Flugsrud et al. [9] reaching 90% (95% CI: 55–100%). No subgroup parameter reached statistical significance for minor complications (Table 5).

**Table 5:** Subgroup analysis of pooled minor complications

Variable	Number of Studies	Proportion	95% Confidence Interval	*p*-value
**Gender**				0.107
Male	21	0.121	0.002–0.059	
Female	24	0.518	0.019–0.131	
**Age**				0.105
≥ 65 years	30	0.044	0.016–0.114	
< 65 years	14	0.009	0.001–0.058	
**Indication**				0.671
Infection	5	0.062	0.000–0.944	
Aseptic Loosening	40	0.027	0.012–0.061	
**Follow-up**				0.074
≥ 5 years	25	0.013	0.003–0.056	
< 5 years	20	0.060	0.022–0.152	
**Paprosky**				1.000
Grades I + II	2	0.000	0.000–1.000	
Grades III + IV	5	0.173	0.001–0.981	
**Endo-Klinik**				0.601
Grades I + II	11	0.035	0.013–0.090	
Grades III + IV	15	0.021	0.003–0.128	
**Stem**				0.555
Short	19	0.022	0.009–0.054	
Long	5	0.055	0.001–0.818	
**Cemented**				0.856
Yes	43	0.030	0.013–0.070	
No	2	0.023	0.000–1.000	

#### Periprosthetic fractures

**Fig. 6:**
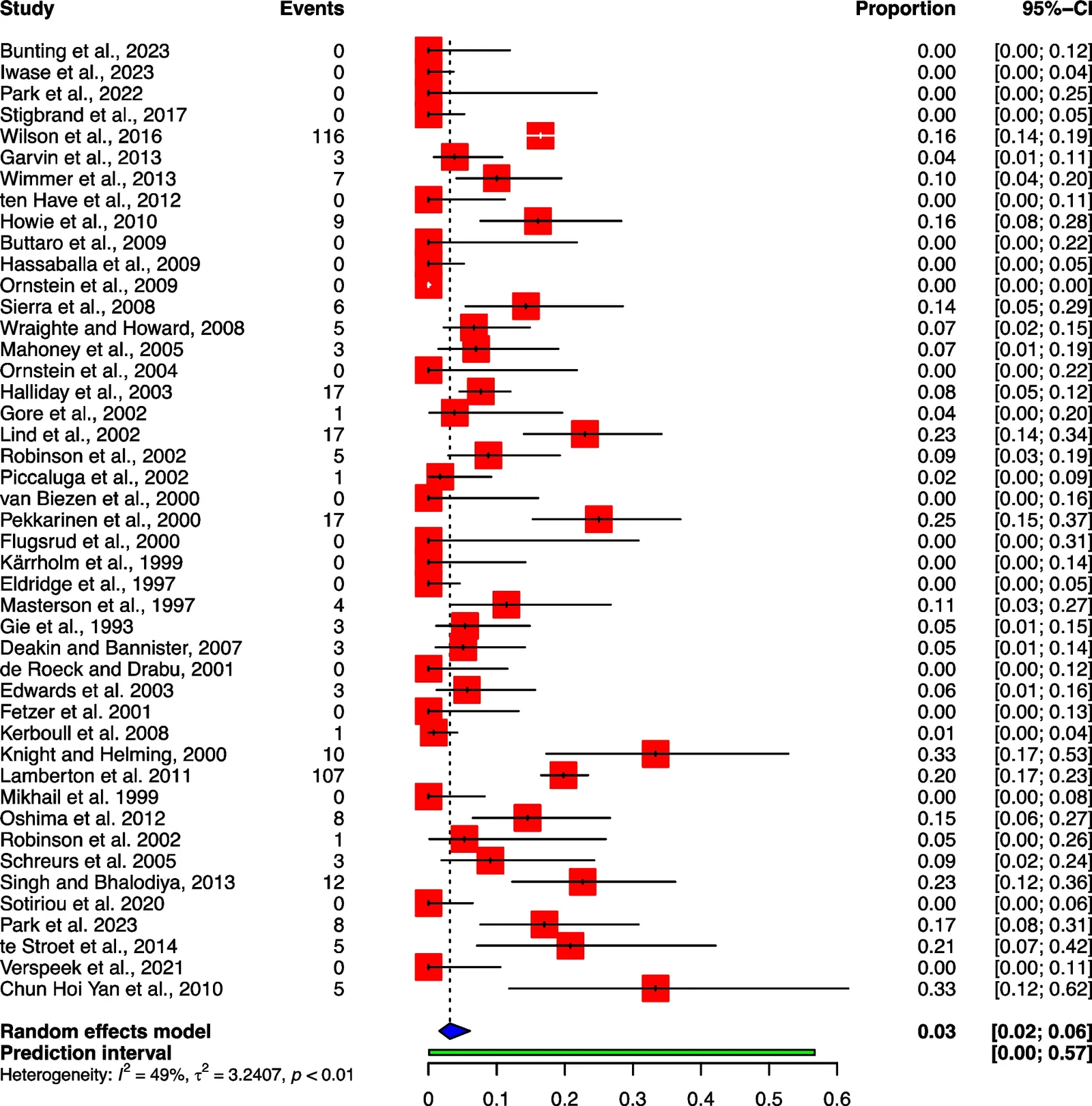
Pooled analysis of intraoperative fractures. The forest plot displays the proportion of intraoperative fractures in each study

The pooled incidence of intraoperative fractures was 3% (95% CI: 2–6%,) with moderate heterogeneity between studies (I^2^ = 49%, *p* < 0.01, Fig. 6). Fractures were more frequently reported in studies using long stems (12.5%, 95% CI: 5.9–24.5%) compared with short stems (2.8%, 95% CI: 0.8–9.2%, *p *= 0.016), and in those using cementless fixation (15.4%, 95% CI: 0.2–93.4%) versus cemented (2.8%, 95% CI: 1.3–5.7%, *p* < 0.001) (Table 6). It should be noted that stem length and fixation method likely serve as proxies for underlying defect severity rather than independent risk factors. No statistically significant difference in intraoperative fracture rates was observed between high- and low-grade bone defects (*p* = 1.0).

**Table 6:** Subgroup analysis of pooled intraoperative periprosthetic fractures

Variable	Number of Studies	Proportion	95% Confidence Interval	*p*-value
**Gender**				0.776
Male	21	0.028	0.010–0.082	
Female	24	0.034	0.014–0.083	
**Age**				0.425
≥ 65 years	30	0.025	0.010–0.060	
< 65 years	14	0.423	0.014–0.124	
**Follow-up**				0.475
≥ 5 years	25	0.025	0.008–0.075	
< 5 years	20	0.040	0.018–0.089	
**Paprosky**				1.000
Grades I + II	2	0.000	0.000–1.000	
Grades III + IV	5	0.003	0.000–0.978	
**Endo-Klinik**				0.423
Grades I + II	11	0.066	0.030–0.143	
Grades III + IV	15	0.045	0.021–0.092	
**Stem**				**0.016**
Short	19	0.028	0.008–0.092	
Long	5	0.125	0.059–0.245	
**Cemented**				** < 0.001**
Yes	43	0.028	0.013–0.057	
No	2	0.154	0.002–0.934	

**Fig. 7:**
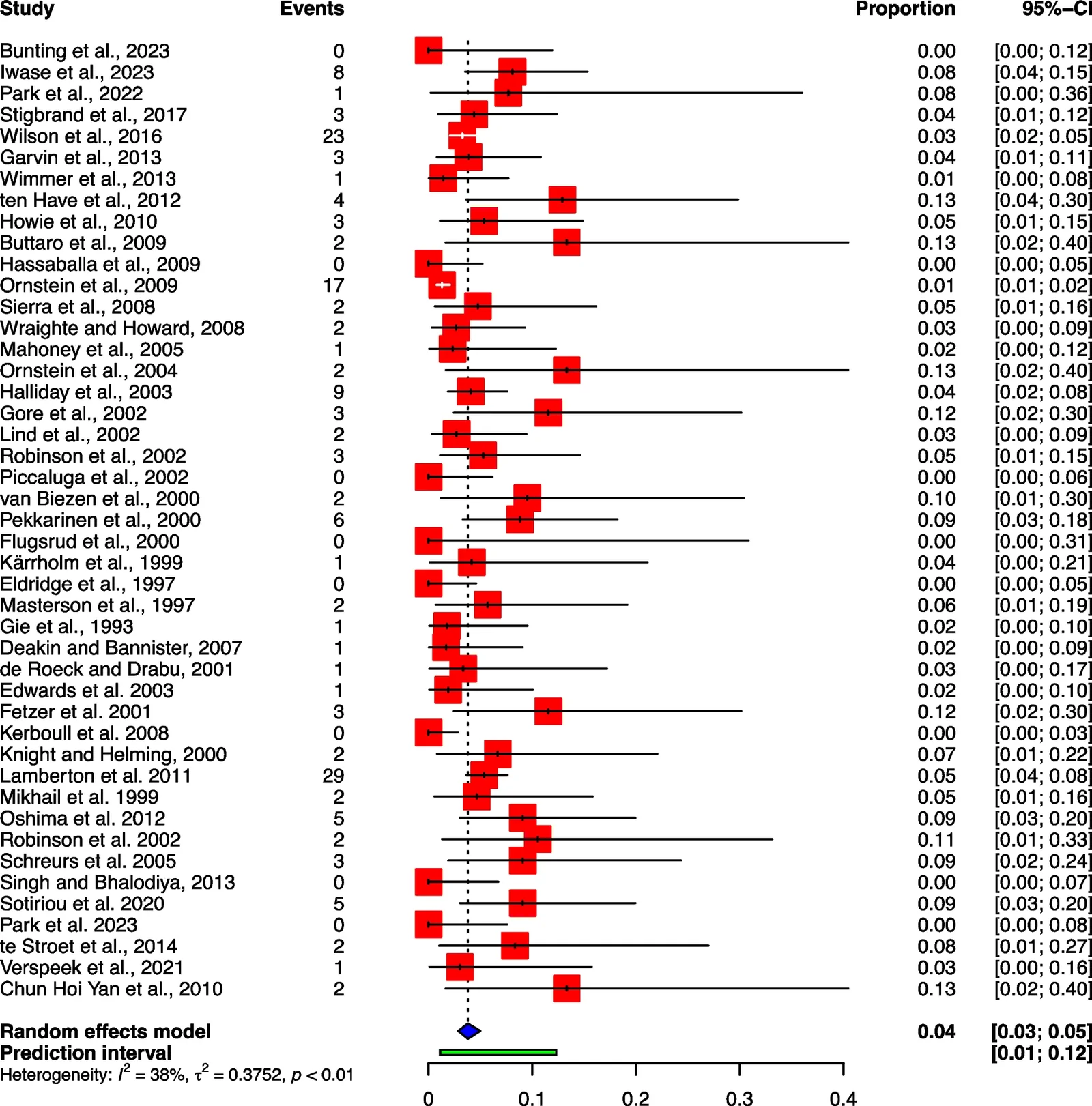
Pooled analysis of postoperative periprosthetic fractures. The forest plot displays the proportion of postoperative periprosthetic fractures in each study

The pooled incidence of postoperative periprosthetic fractures was 4% (95% CI: 3–5%), significantly higher in studies using long stems (4.2%, 95% CI: 2.1–8.4%) compared to short stems (4.1%, 95% CI: 2.7–6.0%, *p* = 0.024), with low heterogeneity between studies (I^2^ = 38%, *p* < 0.01, Fig. 7). There was a trend toward higher fracture incidence in patients ≥ 65 years (4.2%, 95% CI: 3.1–5.7%) versus < 65 years (2.7%, 95% CI: 1.2–6.0%, *p* = 0.267), and in those with grade III/IV bone loss according to Paprosky (4.6% (95% CI: 0.8–23.0%) versus 3.1% (95% CI: 0.0–98.2%), *p *= 0.646) or Endo-Klinik (4.7% (95% CI: 3.1–7.2%) versus 2.3% (95% CI: 0.9–5.7%), *p* = 0.116), though none of these reached statistical significance. The choice of a cemented versus non-cemented stem did not significantly affect the incidence of these fractures (p > 0.05, Table 7).In the pooled analyses (Figs. 4, 5, 6, and 7), squares represent individual studies and horizontal lines their 95% confidence intervals (CIs). Diamonds indicate pooled estimates. I2 reflects between-study heterogeneity, τ^2^ the between-study variance, and prediction intervals the expected range of effects in future studies.

**Table 7:** Subgroup analysis of pooled postoperative periprosthetic fractures

Variable	Number of Studies	Proportion	95% Confidence Interval	*p*-value
**Gender**				0.391
Male	21	0.033	0.021–0.052	
Female	24	0.042	0.029–0.061	
**Age**				0.267
≥ 65 years	30	0.042	0.031–0.057	
< 65 years	14	0.027	0.012–0.060	
**Follow-up**				0.453
≥ 5 years	25	0.041	0.029–0.059	
< 5 years	20	0.033	0.020–0.055	
**Paprosky**				0.646
Grades I + II	2	0.031	0.000–0.982	
Grades III + IV	5	0.046	0.008–0.230	
**Endo-Klinik**				0.116
Grades I + II	11	0.023	0.009–0.057	
Grades III + IV	15	0.047	0.031–0.072	
**Stem**				**0.024**
Short	19	0.041	0.027–0.060	
Long	5	0.042	0.021–0.084	
**Cemented**				0.109
Yes	43	0.040	0.030–0.053	
No	2	0.008	0.000–1.000	

## Discussion

The pooled incidence of major complications in this review was 21% (95% CI: 16%–26%), highlighting the technical demands and morbidity associated with fIBG. The most frequent major complications were intraoperative fractures requiring intervention (23%), followed by postoperative periprosthetic fractures (19%) and instability (19%).

Revision THA carries a higher risk of complications than primary procedures due to poor bone quality, altered anatomy, compromised blood supply and the need for extensive implant and cement removal [62]. These factors increase the risk of biological and mechanical complications during fIBG. Dense sclerotic bone, wide metaphyseal defects and reduced cortical support also reduce tolerance to impaction forces, increasing the risk of fracture or subsidence [63].

There is limited literature comparing complication rates in rTHA across different techniques. Lie et al. reported that nearly half of 4,762 rTHAs involved direct recementation, which yielded inferior outcomes compared to IBG with cementation or uncemented revision [64]. Dislocation incidences following rTHA range from 7 to 20%, consistent with our findings [65]. In one study, female sex was associated with a 71% higher risk of dislocation, which the authors speculated may relate to smaller femoral heads [65]. However, in our analysis we did not observe a sex-based association. Springer et al. identified instability as the leading cause of rTHA failure (35%), whereas periprosthetic fractures accounted for only 2% of re-revisions [66]. In contrast, many of the periprosthetic fractures in the analysis were traumatic rather than technique-related, warranting cautious interpretation of the overall complication incidence.

Infections were the most frequent complication in the study by Badarudeen et al., occurring in 13.8% at three months and 17.3% at one year [67]. Jafari et al. reported an even higher rate (30%) [68]. Our analysis found an infection incidence of 15% among major complications. Higher infection rates in revision surgery likely reflect the biological disruption associated with fIBG, including longer operative times, extensive debridement, greater soft-tissue handling, and the presence of devitalised bone or residual cement. Large defects requiring substantial graft volumes may also delay revascularisation and create a temporary nidus for bacterial colonisation. In addition, many revision patients have undergone multiple prior procedures or have compromised host status, further increasing the risk of infection.

Severe preoperative bone loss (Endo-Klinik grades III–IV) was significantly associated with higher complication incidence (25.3% vs. 14.2%). This is consistent with prior studies [24, 60, 69]. For instance, Pekkarinen et al. found most complications and reoperations occurred in hips with grade III–IV defects [6]. Similar findings were reported by Wilson et al., Ten Have et al., and Garcia-Cimbrelo et al., all noting worse outcomes in patients with advanced bone loss [24, 27, 70]. Nonetheless, Wilson et al. also showed good survival in these cases with precise reconstruction and preserved vascularity [24]. Sierra et al. suggested that improved expertise and tools have enhanced outcomes, even among patients with severe defects [31].

Our analysis found a significantly higher complication incidence with long stems (44.1%) versus short stems (18.8%). A similar pattern was observed for intraoperative fractures, which made up 23% of major complications. The higher fracture rate associated with long stems is likely due to the greater leverage and torsional forces applied during impaction and insertion. Long uncemented stems require distal fixation, necessitating more aggressive canal preparation and higher impaction forces. These factors increase the risk of cortical perforation or fracturing, particularly in widened or osteoporotic canals, which are common in revision settings [71]. In contrast, short cemented stems distribute forces more evenly and rely less on distal bone quality, which may partly explain their lower fracture incidence. However, these associations should be interpreted cautiously, as stem length and fixation method likely reflect underlying defect severity rather than independent risk factors, and subgroup analyses were conducted at the study rather than the patient level. Therefore, higher complication rates with long stems are likely influenced by confounding, and short stems are not necessarily preferable when long stems are clinically indicated.

Lamberton et al. argued that intraoperative complications are more related to the inherent complexity of revision surgery than to fIBG specifically [2]. Stronger meshes and structural allografts have reduced fracture risk in cases with cortical defects or thin bone [72]. However, the impaction process itself, particularly under high compression for stem stability, increases fracture risk [51, 73]. Cortical strut allografts may improve stability in patients with significant bone loss [5].

Stem length significantly influenced intraoperative fracture incidence: long stems were linked to 12.5% of fractures compared to 2.8% with short stems. Although long stems are thought to provide better stability by bypassing cortical defects, this must be weighed against their higher fracture risk [25, 41]. Most short stems in the current review were cemented, designed for controlled subsidence, contrasting with the distal fixation of uncemented stems [74].

While fIBG was originally described with cemented stems[42], both cemented and cementless approaches are now used, with mixed outcomes. In our subgroup analysis, cemented stems had a significantly lower intraoperative fracture rate (2.8% vs. 15.4%). The inclusion of both cemented and cementless techniques introduces inherent heterogeneity, as these approaches differ markedly in their biomechanics, preparation, and expected behavior [75]. Therefore, our subgroup findings reflect associations within heterogeneous groups rather than direct comparisons between equivalent techniques.

Wimmer et al. and Singh et al. reported high intraoperative fracture rates with cementless stems (10% and 23%, respectively), though Singh et al. attributed all fractures to prosthesis and cement removal, not grafting [26, 52]. Some authors suggest fractures are more commonly due to cement extraction[76], while others link them to the impaction process itself. Prophylactic wiring or use of mesh/struts has been recommended in cases with compromised cortex [45]. Releasing tight soft tissues may reduce impaction-related fractures by minimizing bending and torsional stresses [31]. A transtrochanteric approach may also facilitate safe cement removal [46]. Evidence for cementless fIBG remains limited and requires further study.

High intraoperative fracture rates (33%) were reported by authors who used strut grafts for additional fixation, resulting in lower postoperative fracture rates (7% and 13%) [47, 57]. This supports using struts in cases of intraoperative cortical compromise. Garcia-Cimbrelo et al. also used longer stems and cortical struts to reduce fracture risk [70]. Park et al. reported successful fracture healing following strut application, while Wilson et al. noted improved remodeling with impaction grafts [24, 54]. However, concerns remain that extensive cortical onlay grafting may impair femoral blood supply and graft revascularisation [77].

Several limitations affect this review. Heterogeneity in patient data, technique, and implant type – reflected by high I^2^ values – limits generalisability and results in imprecise random-effects estimates, requiring cautious interpretation. Many studies lacked standardised reporting, comparators, prospective data collection, or detailed follow-up. Important confounding variables – including age, comorbidities, surgical indication, fixation method, and implant design – were inconsistently reported and rarely controlled for. Limited reporting on key variables further restricted subgroup analyses. Several studies did not distinguish complications related to implant removal or acetabular exposure from those related to the fIBG itself, which may slightly overestimate technique-related complication rates. Additionally, selection bias is possible, as patients with more severe femoral bone loss were more likely to undergo fIBG and may have a higher baseline risk of complications. Publication bias also cannot be excluded, particularly given the predominance of small case series. Finally, few studies offered rigorous comparisons of fIBG with alternative revision methods.

## Conclusion

The pooled analysis suggested a high incidence of major surgical complications (21%) following fIBG in rTHA, with 78% requiring further surgical intervention. Higher complication rates were reported in cases with severe bone loss (Endo-Klinik grades III and IV) and long stems. However, these findings should be interpreted with caution given the substantial heterogeneity between studies and the fact that subgroup analyses were conducted at the study rather than patient level. Stem length and fixation method likely reflect underlying defect severity rather than independent risk factors. The most common major complications were intraoperative fractures (22.7%), postoperative fractures (19.3%), and instability (19.2%). Fractures were more frequently reported in studies using long and/or cementless stems. Most postoperative fractures occurred more than 12 months after surgery and were commonly associated with trauma rather than technical failure of fIBG.

## Data Availability

The data used in this study are derived from previously published articles, all of which are cited in the manuscript.
